# External validation of the RISC, RISC-Malawi, and PERCH clinical prediction rules to identify risk of death in children hospitalized with pneumonia

**DOI:** 10.7189/jogh.11.04062

**Published:** 2021-10-09

**Authors:** Chris A Rees, Shubhada Hooli, Carina King, Eric D McCollum, Tim Colbourn, Norman Lufesi, Charles Mwansambo, Marzia Lazzerini, Shabir Ahmed Madhi, Clare Cutland, Marta Nunes, Bradford D Gessner, Sudha Basnet, Cissy B Kartasasmita, Joseph L Mathew, Syed Mohammad Akram uz Zaman, Glaucia Paranhos-Baccala, Shinjini Bhatnagar, Nitya Wadhwa, Rakesh Lodha, Satinder Aneja, Mathuram Santosham, Valentina S Picot, Mariam Sylla, Shally Awasthi, Ashish Bavdekar, Jean-William Pape, Vanessa Rouzier, Monidarin Chou, Mala Rakoto-Andrianarivelo, Jianwei Wang, Pagbajabyn Nymadawa, Philippe Vanhems, Graciela Russomando, Rai Asghar, Salem Banajeh, Imran Iqbal, William MacLeod, Irene Maulen-Radovan, Greta Mino, Samir Saha, Sunit Singhi, Donald M Thea, Alexey W Clara, Harry Campbell, Harish Nair, Jennifer Falconer, Linda J Williams, Margaret Horne, Tor Strand, Shamim A Qazi, Yasir B Nisar, Mark I Neuman, World Health Organization PREPARE study group

**Affiliations:** 1 Division of Pediatric Emergency Medicine, Emory University School of Medicine, Children’s Healthcare of Atlanta, Atlanta, Georgia, USA; 2 Section of Pediatric Emergency Medicine, Texas Children’s Hospital, Baylor College of Medicine, Houston, Texas, USA; 3 Department of Global Public Health, Karolinska Institutet, Stockholm, Sweden and Institute for Global Health, University College London, London, UK; 4 Global Program in Respiratory Sciences, Eudowood Division of Pediatric Respiratory Sciences, Department of Pediatrics, Johns Hopkins School of Medicine, Baltimore, USA and Department of International Health, Johns Hopkins Bloomberg School of Public Health, Baltimore, USA; 5 Institute for Global Health, University College London, London, UK; 6 Ministry of Health, Lilongwe, Malawi; 7 WHO Collaborating Centre for Maternal and Child Health, Institute for Maternal and Child Health IRCCS Burlo Garofolo, Trieste, Italy; 8 South African Medical Research Council: Vaccines and Infectious Diseases Analytics Research Unit, School of Pathology, Faculty of Health Sciences, University of the Witwatersrand, Johannesburg, South Africa; Department of Science and Technology/National Research Foundation: Vaccine Preventable Diseases Unit, University of the Witwatersrand, Johannesburg, South Africa; 9 Pfizer Vaccines, Collegeville, Pennsylvania, USA; 10 Center for Intervention Science in Maternal and Child Health, University of Bergen, Norway; 11 Department of Child Health, Faculty of Medicine, Universitas Padjadjaran, Bandung, Indonesia; 12 Pediatric Pulmonology, Postgraduate Institute of Medical Education and Research, Chandigarh, India; 13 Liverpool School of Tropical Medicine, Liverpool, UK; 14 Fondation Merieux, Lyon, France; 15 Translational Health Science and Technology Institute, Faridabad, India; 16 All India Institute of Medical Sciences, New Delhi, India; 17 School of Medical Sciences & Research, Sharda University, Greater Noida, India; 18 Department of International Health, Bloomberg School of Public Health, Johns Hopkins University, Baltimore, Maryland, USA; 19 Fondation Merieux, Lyon, France; 20 Gabriel Touré Hospital, Department of Pediatrics, Bamako, Mali; 21 King George’s Medical University, UP, Department of Pediatrics, Lucknow, India; 22 KEM Hospital Pune, Department of Pediatrics, Pune, India; 23 GHESKIO, Centre GHESKIO, Port au Prince, Haiti; 24 GHESKIO, Department of Pediatrics, Port au Prince, Haiti; 25 University of Health Sciences Faculty of Medicine, Rodolph Mérieux Laboratory, Phom Phen, Cambodia; 26 Centre d'Infectiologie Charles Mérieux, Antanarivo, Madagascar; 27 Chinese Academy of Medical Sciences & Peking Union, Medical College Institute of Pathogen Biology, MOH Key Laboratory of Systems Biology of Pathogens and Dr Christophe Mérieux Laboratory, Beijing, China; 28 Mongolian Academy of Sciences, Academy of Medical Sciences, Ulaanbaatar, Mongolia; 29 Hospices Civils de Lyon, Infection Control Unit; CIRI, Centre International de Recherche en Infectiologie, (Team PHE3ID), Université Claude Bernard Lyon, Lyon, France; 30 Universidad Nacional de Asuncion, Instituto de Investigaciones en Ciencias de la Salud, San Lorenzo, Paraguay; 31 Rawalpindi Medical College, Rawalpindi, Pakistan; 32 Sana’a University, Sana’a, Yemen; 33 Nishtar Medical College, Multan, Pakistan; 34 Department of Global Health, Boston University School of Public Health, Boston, Massachusetts, USA; 35 Instituto Nactional de Pediatria Division de Investigacion Insurgentes, Mexico City, Mexico; 36 Children's Hospital Dr Francisco de Ycaza Bustamante, Head of Department, Infectious diseases, Guayaquil, Ecuador; 37 Dhaka Shishu Hospital, Dhaka, Bangladesh; 38 Medanta, The Medicity, Gurgaon, India; 39 Department of Global Health, Boston University School of Public Health, Boston, Massachusetts, USA; 40 US Centers for Disease Control, Central American Region, Guatemala City, Guatemala; 41 Centre for Global Health, Usher Institute, The University of Edinburgh, Edinburgh, Scotland; 42 Institute for Global Health and Development, Queen Margaret University, Edinburgh, Scotland; 43 Centre for Global Health, Usher Institute, The University of Edinburgh, Edinburgh, Scotland; 44 Research Department, Innlandet Hospital Trust, Lillehammer, Norway; 45 Department of Maternal, Newborn, Child and Adolescent Health (Retired), World Health Organization, Geneva, Switzerland; 46 Department of Maternal, Newborn, Child and Adolescent Health and Ageing, World Health Organization, Geneva, Switzerland; 47 Division of Emergency Medicine, Boston Children’s Hospital, Harvard Medical School, Boston, Massachusetts, USA; 48 Pneumonia Research Partnership to Assess WHO Recommendations

## Abstract

**Background:**

Existing scores to identify children at risk of hospitalized pneumonia-related mortality lack broad external validation. Our objective was to externally validate three such risk scores.

**Methods:**

We applied the Respiratory Index of Severity in Children (RISC) for HIV-negative children, the RISC-Malawi, and the Pneumonia Etiology Research for Child Health (PERCH) scores to hospitalized children in the Pneumonia REsearch Partnerships to Assess WHO REcommendations (PREPARE) data set. The PREPARE data set includes pooled data from 41 studies on pediatric pneumonia from across the world. We calculated test characteristics and the area under the curve (AUC) for each of these clinical prediction rules.

**Results:**

The RISC score for HIV-negative children was applied to 3574 children 0-24 months and demonstrated poor discriminatory ability (AUC = 0.66, 95% confidence interval (CI) = 0.58-0.73) in the identification of children at risk of hospitalized pneumonia-related mortality. The RISC-Malawi score had fair discriminatory value (AUC = 0.75, 95% CI = 0.74-0.77) among 17 864 children 2-59 months. The PERCH score was applied to 732 children 1-59 months and also demonstrated poor discriminatory value (AUC = 0.55, 95% CI = 0.37-0.73).

**Conclusions:**

In a large external application of the RISC, RISC-Malawi, and PERCH scores, a substantial number of children were misclassified for their risk of hospitalized pneumonia-related mortality. Although pneumonia risk scores have performed well among the cohorts in which they were derived, their performance diminished when externally applied. A generalizable risk assessment tool with higher sensitivity and specificity to identify children at risk of hospitalized pneumonia-related mortality may be needed. Such a generalizable risk assessment tool would need context-specific validation prior to implementation in that setting.

Worldwide, pneumonia is the most common cause of mortality among children 1-59 months old [1,2]. Despite dramatic reductions in under-five mortality rates during the Millennium Development Goal era, pneumonia caused more than 800 000 pediatric deaths as recently as 2017 [2,3]. Identification of children at risk of pneumonia-related mortality is the first step in directing supportive treatments, hospitalization, and oxygen support that have the potential to reduce these deaths. Clinical prediction rules, which take into account clinical data to predict an outcome, may aid in the distribution of resources to children at greatest risk of hospitalized pneumonia-related mortality.

To date, four clinical prediction rules have been derived to identify children at risk of hospitalized pneumonia-related mortality [4-7]. The Respiratory Index of Severity in Children (RISC) score was developed retrospectively from >4000 hospitalized children aged 0-24 months old, with nearly 300 deaths, in South Africa in a 9-valent pneumococcal conjugate vaccine trial between 1998 and 2001 [4]. The RISC score stratified children who were human immunodeficiency virus (HIV)-positive and those who were HIV-negative. The Modified Respiratory Index of Severity in Children (mRISC) score was developed in >3500 hospitalized children aged 0-59 months with >200 deaths in Kenya from 2009 to 2012 [5]. The third score, the RISC-Malawi score, was developed retrospectively using routinely collected clinical data from a cohort of >14 000 hospitalized children 2-59 months old in Malawi, with more than 400 deaths from 2011 to 2014 [6]. Most recently, the Pneumonia Etiology Research for Child Health (PERCH) group derived a scoring system from 1800 HIV-negative children aged 1-59 months with 120 deaths from seven countries: Kenya, the Gambia, Mali, Zambia, South Africa, Thailand and Bangladesh [7]. The PERCH study aimed to determine the etiology of pneumonia among children presenting to hospitals in these regions from 2011-2014 [7].

These risk scores have not been widely validated using data from low- and middle-income settings outside sub-Saharan Africa [8,9]. The RISC score for HIV-negative children had fair discriminatory value when retrospectively applied to the PERCH and RISC-Malawi data sets, both of which were done after the pneumococcal vaccine became available and large scale prevention of mother to child transmission of HIV implementation [6,7]. The mRISC score, which used non-standard variables, to our knowledge has not been externally validated in any setting. In order for clinical prediction rules to be widely accepted and utilized, they must incorporate variables that can be feasibly collected in clinical practice.

Given the lack of published reports of their implementation, the impact of existing clinical prediction rules on reducing hospitalized pneumonia-related mortality is unclear [10]. Broad validation of existing clinical prediction rules to identify children at risk of hospitalized pneumonia-related mortality in diverse settings is the first step towards wider clinical application of useful and robust clinical prediction rules. To this end, we aimed to externally validate existing clinical prediction rules for hospitalized pneumonia-related mortality in a diverse cohort of hospitalized children from the World Health Organization’s (WHO) Pneumonia REsearch Partnership to Assess WHO REcommendations (PREPARE) study group.

## METHODS

### Study design

We applied the RISC, RISC-Malawi, and PERCH scores for hospitalized pneumonia-related mortality to all hospitalized children with all included parameters in the WHO PREPARE study group data set. The mRISC score includes several variables (ie, history of night sweats, dehydration, history of decreased consciousness, and history of inability to drink or breastfeed) which were not widely available in the WHO PREPARE study group data set. Thus, similar to prior work attempting to externally validate the mRISC score [6], we did not include it in our analysis. We were also unable to externally apply the RISC score for HIV-positive children as it already been applied to 152 of the 159 children who had all reported parameters in the WHO PREPARE data set [6].

### Ethical considerations

This study used de-identified individual patient data from previously published studies in which ethical approval was obtained at each participating site. Additional ethical approval was obtained by the WHO ethics review committee for one study sponsored by the WHO.

### Data source

The WHO PREPARE data set has been described previously [11]. Briefly, this data set includes primary, patient-level data for children 0-59 months old evaluated for pneumonia. This is from 30 diverse study groups, comprising 41 separate data sets, from over 20 low- and middle-income countries in Asia, Africa, and Latin America as well as the United States of America and Australia. Data sets were identified from a systematic review of childhood pneumonia [12]. Several additional data sets were included in PREPARE as the WHO was aware of ongoing and relevant studies. Investigators for these additional studies were invited to contribute to the PREPARE data set.

Although some data sets included older children, the cohort was restricted to children 0-59 months of age to be consistent with most other pneumonia investigations in children [13-15]. We excluded community-based studies because our outcome was hospital based. We also excluded hospital-based studies that did not report survival data.

Pneumonia was defined in included studies included in the WHO PREPARE data set according to the WHO Pocket Book of Hospital Care for Children, based on the presence of age-adjusted tachypnea, lower chest indrawing, general danger signs (eg, abnormally sleepy, lethargy, central cyanosis, inability to drink, or convulsions), or signs of respiratory distress (eg, head nodding/bobbing, nasal flaring, or grunting) in children with a cough or difficulty breathing [16].

### Variables

Our outcome measure was hospitalized pneumonia-related mortality during the respective study periods. Variables included in the RISC, RISC-Malawi, and PERCH scores are found in **Table 1**. The RISC score for HIV-negative children had a range of -2 to 6 points [4]. All variables included in the original RISC score for HIV-negative children were included in this external application. The RISC score assigned 3 points to HIV-negative children with SpO_2_≤90%. However, if a child’s SpO_2_ was >90%, chest indrawing became a part of the scoring system. If the SpO_2_ was ≤90%, chest indrawing was not included in the score assignment. Though originally derived among children 0-24 months old, we also applied the RISC score for HIV-negative children to children 0-59 months old who had all necessary parameters available as a supplement.

**Table 1:** Summary of variables included in the Respiratory Index of Severity in Children (RISC) [4], RISC-Malawi [6], and Pneumonia Etiology Research for Child Health (PERCH) [7] hospitalized pneumonia-related mortality prediction scores

Parameter	Weighted points	Original derivation area under the curve
**RISC for HIV-negative children 0-24 months**		
Oxygen saturation (SpO_2_)≤90%	3	0.92
*OR*		
Chest indrawing	2	
Wheezing	-2	
Refusal to feed	1	
World Health Organization (WHO) weight for age z-score≤-3	2	
WHO weight for age z-score>-3 to≤-2	1	
**Maximum score (original and external application)**	**6**	
**RISC-Malawi using weight for age z-score for children 2-59 months**		
SpO_2_≥93%	0	0.80
SpO_2_ 90%-92%	1	
SpO_2_<90%	5	
WHO weight for age z-score≥-2	0	
WHO weight for age z-score≥-3 to<-2	3	
WHO weight for age z-score<-3	6	
Male	0	
Female	1	
Wheezing	-1	
Unconscious	5	
**Maximum score (original and external application)**	**17**	
**PERCH Study score for HIV-negative children 1-59 months**		
Age 1-11 months	2	0.76
Age 12-59 months	0	
Male	0	
Female	1	
Unresponsive without deep breathing*	2	
Unresponsive with deep breathing*	5	
Cough (observed)	-1	
Grunting (observed)	2	
SpO_2_<92%	2	
Maximum duration of illness 3-5 d	2	
Maximum duration of illness >5 d	2	
WHO weight for height z-score≥-2†	0	
WHO weight for height z-score≥-3 to<-2	2	
WHO weight for height z-score<-3	3	
**Maximum score (original)**	**17**	
**Maximum score (external application)**	**12**	

*Deep breathing not available in WHO PREPARE data set.

†Weight for age z-score used in the external application.

The RISC-Malawi score had a range of -1 to 17 using weight-for-age z-score (WAZ) and a range of -2 to 23 points using mid-upper arm circumference (MUAC) [6]. As MUAC was only reported in two studies that met all inclusion criteria within the WHO PREPARE data set [17,18], we used the RISC-Malawi score with WAZ, applied to children aged 2-59 months, for our external application. The RISC-Malawi score using MUAC analysis is included as a supplement. The WHO PREPARE data set contains the RISC-Malawi development data set, and therefore these data were excluded from the external validation of the RISC-Malawi score [6].

The PERCH score had a range of -1 to 17 [7]. Its primary outcome was hospitalized pneumonia-related mortality or death within 7 days of hospital discharge. The PREPARE data set does not include the variable “deep breathing”, which was included in the PERCH study, so this was not included in our analysis. Therefore, the maximum PERCH score in our external validation was 12 instead of 17. Furthermore, the PERCH score included the parameter of observed cough. For our external application, we included history of cough in place of observed cough. We used the variable unconsciousness in place of unresponsiveness as the definitions of both of these included descriptions such as unconsciousness, unresponsiveness, lethargy, and abnormally sleepy. In lieu of weight-for-height, we used WAZ to measure children’s nutritional status as follows: weight-for-height z-score<-3 corresponded to WAZ<-3 for severe acute malnutrition, weight-for-height z-score≥–3 to<–2 corresponded to WAZ of -3 to -2 for moderate acute malnutrition, and weight-for-height z-score≥-2 corresponded to WAZ>-2 for normal weight.

### Data analysis

We determined the accuracy of the RISC, RISC-Malawi, and PERCH scores on hospitalized children 0-59 months old in the WHO PREPARE data set. We calculated the case fatality ratio (CFR), as the number of deaths/number of patients, and the percentage of patients who were correctly classified as true positive and true negative cases among all children at each cut point for each score. We calculated the sensitivity, specificity, and positive and negative likelihood ratios (LR) of each score at ≥ each specified cut point as defined in the scores. We created receiver operating characteristic (ROC) curves for each risk score. Based on published standards, we used the following scale to qualify the discriminatory ability of each score: area under the curve (AUC)≥0.90 for “excellent discrimination”, AUC 0.80 to 0.89 for “good discrimination”, AUC = 0.70-0.79 for “fair discrimination”, and “poor discrimination” for AUC<0.70 [19,20].

We conducted a complete case analysis and excluded cases with missing data for any parameter. In order to accurately identify children at low-risk of hospitalized pneumonia-related mortality, we aimed to report maximum sensitivity without sacrificing specificity. We, therefore, report risk score cut points with the sensitivity of 60%-80% with concurrent specificity of at least 40%. We present risk predictiveness curves, to show the cumulative percentage of children at risk of hospitalized pneumonia-related mortality by their predicted risk. We report test characteristics and 95% confidence intervals (CI) for the +LRs and the -LRs. All analyses were conducted using Stata version 14.2 (Stata-Corp, College Station, TX, USA).

## RESULTS

### Study population

**Figure 1:**
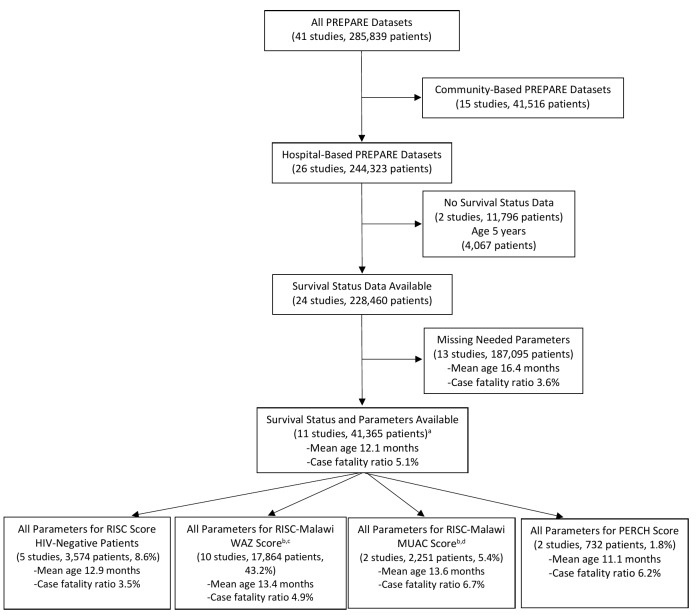
Selection of children aged 0-59 months from hospital-based studies included in the external application of the Respiratory Index of Severity in Children (RISC), RISC-Malawi, and Pneumonia Etiology Research for Child Health (PERCH) scores. ^a^The sum of patients included in the 4 individual validation cohorts does not total 41 365 as the validation each of the 4 scores required that every component of the score be available within the data set. ^b^Excluding the parent data set from which the RISC-Malawi score was derived. ^c^Excluding 610 children less than 2 months of age. ^d^Excluding 105 children less than 2 months of age.

Of the 41 separate data sets in the PREPARE study, there were 26 hospital-based studies and 15 community-based studies (**Figure 1**). Of the 26 hospital-based studies in the WHO PREPARE data set, 24 studies included data on patients’ survival status through hospitalization, with 228 460 patients aged 0-59 months old who were evaluated for pneumonia. Among these, there were 8820 (3.9%) hospitalized pneumonia-related deaths. We were able to apply the RISC score for HIV-negative children 0-24 months old to 3574 children from five studies [6,17,18,21,22], the RISC-Malawi score to 17 864 children 2-59 months old from 10 studies [17,18,21-28], and the PERCH score to 732 children 1-59 months old from two studies [17,21] (**Figure 1**). A total of 732 children had data available for all three scores [17,21]. Characteristics of individual included studies are found in **Table 2**. The CFR in the included studies ranged from 0.9%-10.9%.

**Table 2:** Characteristics of studies included in the Pneumonia REsearch Partnerships to Assess WHO REcommendations (PREPARE) data set

Study	Location	Study design	Age range (months)	Sample size, N	Deaths, n (%)	Inclusion and exclusion criteria	Score(s) applied (n)
Hooli 2016 [6]	Mchinji and Lilongwe Districts, Malawi	Prospective cohort	0-59	16 475	524 (3.2)	Inclusion: children with World Health Organization (WHO)-defined pneumonia* of any severity	Respiratory Index of Severity in Children (RISC) HIV-negative (2435)
Mathew 2015 [17]	Chandigarh, India	Prospective cohort	1-59	2400	157 (6.5)	Inclusion: children with WHO-defined pneumonia* of any severity	RISC HIV-negative (104)
							RISC-Malawi (1,689)
							Pneumonia Etiology Research for Child Health (PERCH) (128)
Benet 2017 [18]	Phnom Penh, Cambodia	Prospective, case-control study	2-59	888	20 (2.2)	Inclusion for cases: Children with WHO-defined pneumonia with first symptoms lasting <14 days	RISC HIV-negative (445)
	Beijing, China						
	Port au Prince, Haiti						
	Lucknow, India						
	Pune-Vadu, India						
	Antananarivo, Madagascar						
	Banako, Mali					Radiographic confirmation of pneumonia (WHO criteria)	
	Ulaanbaatar, Mongolia					No wheezing	
	San Lorenzo, Paraguay					Inclusion for controls: no symptoms suggestive of respiratory illness, outpatients, or hospitalized in surgery	RISC-Malawi (615)
Basnet 2012 [21]	Kathmandu, Nepal	Randomized controlled trial	2-35	641	6 (0.9)	Inclusion: children with a complaint of cough for <14 days, complaint of difficulty breathing for ≤72 h, presence of lower chest wall indrawing, or presence of lower chest indrawing on examination by study physician for severe pneumonia	RISC HIV-negative (587)
							RISC-Malawi (604)
							PERCH (604)
Clara [22]	Chiriqui Province, Panama	Retrospective cohort	0-59	85	2 (2.3)	Inclusion: children with severe acute respiratory infection case definition	RISC HIV-negative (3)
							RISC-Malawi (37)
Gessner 2005 [23]	Lombok, Indonesia	Hamlet-randomized trial	0-23	6221	676 (10.9)	Inclusion: children with WHO-defined pneumonia	RISC-Malawi (5,807)
Klugman 2003 [24]	Johannesburg, South Africa	Randomized controlled trial	0-59	10 114	452 (4.5)	Inclusion: based on International Classification of Diseases (ICD)-10 codes for pneumonia	RISC-Malawi (7,283)
Asghar 2008 [25]	Dhaka, Bangladesh	Randomized controlled trial	2-59	958	46 (4.8)	Inclusion: children with WHO-defined very severe pneumonia†	RISC-Malawi (802)
	Chandigarh, India						
	Mexico City, Mexico						
	Rawalpindi, Pakistan						
	Multan, Pakistan						
	Sana’a, Yemen						
	Lusaka, Zambia						
Wadhwa 2013 [26]	New Delhi, India	Randomized controlled trial	2-24	550	8 (1.5)	Severe pneumonia: fast breathing: ≥50 breaths/min in children 2-11 months &≥40 breaths/min in children 12-24 months	RISC-Malawi (461)
						Crepitations on auscultation	
						Presence of chest indrawing	
						Very severe pneumonia: severe pneumonia (with or without chest indrawing) and any general danger sign (ie, lethargy or inability to drink or convulsions) or central cyanosis	
Cutts 2005. [27]	Upper River and Central River Regions, The Gambia	Randomized controlled trial	1-30	1716	106 (6.2)	Inclusion: history of cough or difficult breathing with an elevated respiratory rate for age (≥50 breaths per min for children <1 year old and ≥40 per min for children ≥1 year old) or lower chest-wall indrawing	RISC-Malawi (346)
Wulandari 2018 [28]	West Java, Indonesia	Retrospective cohort	0-59	1317	69 (5.2)	Inclusion: children with WHO-defined pneumonia	RISC-Malawi (830)

*WHO-defined pneumonia: presence of age-specific fast breathing, lower chest indrawing, or general danger signs in children with a cough or difficulty

†Cough and/or difficulty breathing, and central cyanosis or inability to drink.

### RISC score for HIV-negative children

**Figure 2:**
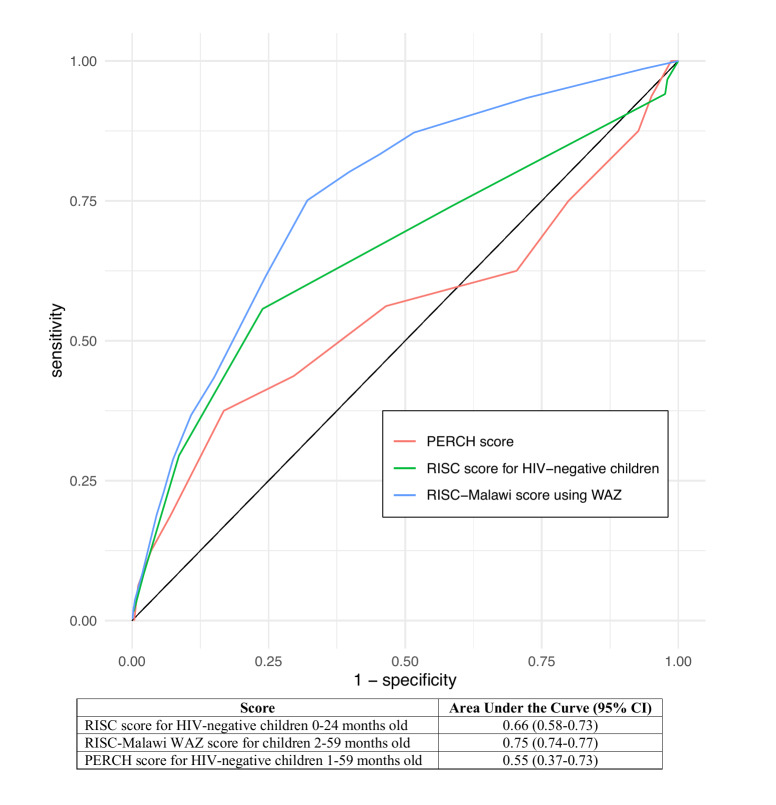
Receiver Operating Characteristic (ROC) curve for the Respiratory Index of Severity in Children (RISC) score for HIV-negative children 0-24 months old, RISC-Malawi weight-for-age (WAZ) score for children 2-59 months old, and Pneumonia Etiology Research for Child Health **(**PERCH) score for HIV-negative children 1-59 months old for hospitalized pneumonia-related mortality.

The numbers of patients who had each parameter of the RISC score for HIV-negative children with corresponding CFRs are found in **Table 3**. Of the parameters in the RISC score for HIV-negative children 0-24 months old, children who refused to feed at presentation had the highest CFR and children who had presented with wheeze had the lowest CFR. The RISC score for HIV-negative children 0-24 months old had poor discriminatory ability in identifying children at risk of hospitalized pneumonia-related mortality (**Figure 2**). The CFR at a RISC score of 2 out of 6 for HIV-negative children 0-24 months old was 0.8%. Using a score of ≥2, the RISC score for HIV-negative children 0-24 months old had 74.1% sensitivity, 41.4% specificity, +LR of 1.26 (95% CI 1.05-1.44), and -LR of 0.64 (95% CI 0.45-1.01) (Table S1 in the **Online Supplementary Document**). The RISC score for HIV-negative children performed similarly when applied to 4061 children aged 0-59 months with a score of 2 out of 6 (Table S2 and Table S3 in the **Online Supplementary Document**).

**Table 3:** Number of patients, deaths, and case fatality ratio of parameters used in the Respiratory Index of Severity in Children (RISC) score for HIV-negative children 0-24 mo old in the Pneumonia REsearch Partnerships to Assess WHO REcommendations (PREPARE) data set (n = 3574)

Parameter	Sign or symptom present, n (%)	Deaths, n	Case fatality ratio % (95% confidence interval)
Oxygen saturation (SpO_2_)≤90%	917 (25.6)	20	2.2 (1.3-3.3)
Chest indrawing (with SpO_2_>90%)	2358 (66.0)	27	1.1 (0.7-1.7)
Wheezing	1487 (41.6)	15	1.0 (0.6-1.7)
Refusal to feed	351 (9.8)	16	4.6 (2.6-7.3)
World Health Organiztion (WHO) weight for age z-score≤-3	285 (8.0)	10	3.5 (1.7-6.3)
WHO weight for age z-score -2≤z<-3	435 (12.2)	13	3.0 (1.6-5.0)
Total		61	1.7 (1.3-2.2)

#### RISC-Malawi Score

We applied the RISC-Malawi score using WAZ to 17 864 children 2-59 months old in the WHO PREPARE data set. Of the parameters in the RISC-Malawi score, patients who had documented unconsciousness at presentation had the highest CFR, and children with SpO_2_≥93% had the lowest CFR (**Table 4**). The RISC-Malawi score had fair discriminatory value in the identification of children at risk of hospitalized pneumonia-related mortality (**Figure 2**). At a score of ≥5 out of 17, the RISC-Malawi score had 75.1% sensitivity, 67.9% specificity, +LR of 2.27 (95% CI = 2.17-2.37), and -LR of 0.37 (95% CI = 0.33-0.42) in identifying children at risk of hospitalized pneumonia-related mortality (Table S4 in the **Online Supplementary Document**). Using a cut-point score of ≥6 out of 17, the RISC-Malawi score using WAZ had 61.6% sensitivity, 75.5% specificity, +LR of 2.51 (95% CI = 2.37-2.66), and -LR of 0.51 (95% CI = 0.47-0.55) (Table S4 in the **Online Supplementary Document**).

**Table 4:** Number of patients, deaths, and case fatality ratio for parameters in the Respiratory Index of Severity in Children (RISC)-Malawi score using weight-for-age z-score (WAZ) for children 2-59 mo old in the PREPARE data set (n = 17 864)

Parameter	Sign or symptom present, n (%)	Deaths, n	Case fatality ratio % (95% confidence interval)
Oxygen saturation (SpO_2_):			
SpO_2_≥93%	9804 (54.9)	243	2.5 (2.2-2.8)
SpO_2_ 90%-92%	3727 (21.9)	118	3.2 (2.6-3.8)
SpO_2_<90%	4333 (24.2)	508	11.7 (10.8-12.7)
World Health Organization (WHO) weight-for-age z score (WAZ) categories:			
WAZ>-2	11 973 (67.0)	395	3.3 (3.0-3.6)
WAZ -2 to -3	3157 (17.7)	212	6.7 (5.9-7.6)
AZ<-3	2734 (15.3)	262	9.6 (8.5-10.7)
Female	8360 (46.8)	466	5.6 (5.1-6.1)
Wheezing	5489 (30.7)	174	3.2 (2.7-3.7)
Unconscious/decreased consciousness	738 (4.1)	96	13.0 (10.7-15.6)
Total		869	4.9 (4.5-5.2)

When applying the RISC-Malawi MUAC score to 2251 patients, the parameter SpO_2_<90% demonstrated the highest CFR (Table S5 in the **Online Supplementary Document**). Though the +LR and -LR were slightly lower with MUAC included instead of WAZ, overall the RISC-Malawi score performed similarly with MUAC included as the parameter for malnutrition (Table S6 in the **Online Supplementary Document**).

### PERCH score for HIV-negative children

We applied the PERCH score to 732 children 1-59 months old in the WHO PREPARE data set. Of the parameters included in the PERCH score, patients who had WAZ<-3 had the highest CFR. Children with unresponsiveness had the lowest CFR (**Table 5**). The PERCH score had poor discriminatory value in identifying children at risk of hospitalized pneumonia-related mortality (**Figure 2**). The CFR at a PERCH score of 5 was 0.6%. A PERCH score of ≥5 had 62.5% sensitivity, 29.6% specificity, +LR of 0.89 (95% CI = 0.61-1.30), and -LR of 1.27 (95% CI = 0.67-2.41) (Table S7 in the **Online Supplementary Document**).

**Table 5:** Number of patients, deaths, and case fatality ratio by parameter used in the Pneumonia Etiology Research for Child Health (PERCH) study score for HIV-negative children 1-59 mo old in the Pneumonia REsearch Partnerships to Assess WHO REcommendations (PREPARE) data set (n = 732)

Parameter	Sign or symptom present, n (%)	Deaths, n	Case fatality ratio % (95% confidence interval)
Age 1-11 months	569 (77.7)	13	2.3 (1.2-3.9)
Female	277 (37.8)	7	2.5 (1.0-5.1)
Unresponsiveness	102 (13.9)	0	0.0 (-)
Cough – (history)	731 (99.9)	16	2.2 (1.3-3.5)
Grunting – (observed)	137 (18.7)	5	3.7 (1.2-8.3)
Oxygen saturation (SpO_2_)<92%	526 (71.9)	9	1.7 (0.8-3.2)
Maximum duration of illness – 3 d or more	633 (86.5)	15	2.4 (1.3-3.9)
World Health Organization (WHO) weight-for-age z-score<-3	83 (11.3)	6	7.2 (2.7-15.1)
WHO weight-for-age z-score≥-3 to<-2	120 (16.4)	1	0.8 (0.01-4.6)
Total		16	2.2 (1.2-3.5)

#### Risk predictiveness curves

**Figure 3:**
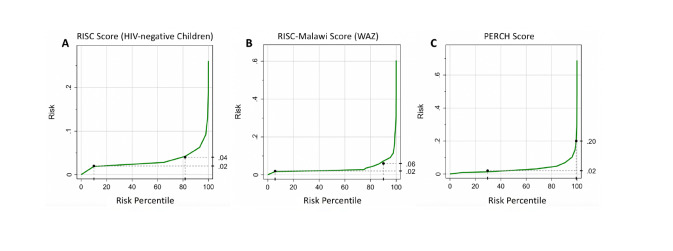
Risk predictiveness curves of the Respiratory Index of Severity in Children (RISC) score for HIV-negative children 0-24 months old (**A**), RISC-Malawi weight-for-age (WAZ) score for children 2-59 months old (**B**), and Pneumonia Etiology Research for Child Health **(**PERCH) score for HIV-negative children 1-59 months old (**C**) for hospitalized pneumonia-related mortality.

Risk predictiveness curves demonstrated that the majority of children were low risk for mortality (**Figure 3**).

## DISCUSSION

Clinical prediction rules previously derived among children in sub-Saharan Africa, Thailand, and Bangladesh performed sub-optimally in identifying hospitalized pneumonia-related mortality when externally applied to a diverse population of children from 19 countries. Both the RISC score for HIV-negative children and the PERCH score had poor discriminatory value when externally applied to the PREPARE data set, and the RISC-Malawi score had fair discriminatory value in identifying children at risk of hospitalized pneumonia-related mortality.

Among HIV-negative children, we observed that the RISC score had poor discriminatory value in identifying children at risk of hospitalized pneumonia-related mortality in both children 0-24 and 0-59 months old. This is in contrast to the external application of the RISC score for HIV-negative among >14 000 children in Malawi and the external application in the PERCH data set including >1800 children from seven countries in which RISC score had fair discriminatory value among all children and among HIV-exposed children [6,7]. The poor performance of the RISC score in our external application may be due to having more diverse patient populations, including differing rates and types of comorbidities among children outside sub-Saharan Africa. Although 68% of patients included in our external application were located in sub-Saharan Africa, these studies generally have lower childhood mortality rates than the original patient group. Given the challenges we found in accessing reliable routine data on HIV-status in children, changes in HIV prevention and management in sub-Saharan Africa [29], and assumed changes in underlying pneumonia etiology since widespread pneumococcal vaccine introduction [30], poor to fair external performance was not necessarily surprising.

Though the PERCH score derivation included multiple countries, unlike the other scores evaluated, it had the poorest discriminatory value in identifying children at risk of hospitalized pneumonia-related mortality. This may be partly explained by our inability to include the variable “deep breathing” or the substitution of WAZ in place of weight-for-height z-score. Deep breathing is not a standard variable in the WHO screening for pneumonia. Similar to the mRISC score, which has not been externally validated, this highlights the challenge of including variables that are not included in routine guidelines in practical clinical prediction rules. While such inclusion may improve local performance, their ability to be pragmatically scaled or validated may be limited. Furthermore, our substitution of WAZ for weight-for-height z-score may also limit the external validation of the PERCH score as weight-for-height z-score may more accurately account for both present and past nutritional status [31]. Further validation studies using the precise variables included in the PERCH score across a variety of settings are warranted.

Among the clinical prediction rules externally applied in our study, the RISC-Malawi score had the most discriminatory value. However, the AUC for the RISC-Malawi score demonstrated only fair discriminatory value. The improved performance of the RISC-Malawi score may be due to fewer physical examination findings included as parameters in this score. Variation in inter-observer agreement of physical examination findings could lead to classification errors that would ultimately under-estimate the effect of a clinical prediction tool [32,33]. Moreover, malaria and HIV, though endemic in Malawi, were not incorporated into the score due to underreporting of these variables [6]. As HIV and malaria are common in Malawi among children [34,35], it is possible that the exclusion of these variables may have driven mortality risk as an unmeasured confounding parameter. Lastly, we were able to apply the RISC-Malawi score to over 17 000 children, which suggests it may be the clinical prediction rule that allows for widest dissemination based on the use of data routinely collected in clinical practice across a variety of settings.

Although clinical prediction rules tend to underperform when applied externally to populations in which they were derived, none of the clinical prediction rules externally applied here demonstrated excellent discriminatory value. Thus, clinical prediction rules for hospitalized pneumonia-related mortality may not be confidently applied in settings beyond where they were derived. Some of the underperformance of these clinical prediction rules in our external validation may be due to varying resources such as high-flow nasal cannula, non-invasive ventilation, or mechanical ventilation and perhaps limited oxygen and pulse oximetry in some settings included in the WHO PREPARE data set. Furthermore, variations in quality of care, diagnostic capabilities, region-specific pathogens implicated in childhood pneumonia, and antibiotic use across the 19 countries included in our analysis may have contributed to the underperformance of these clinical prediction rules.

Age <12 months, chest indrawing, grunting, respiratory rate >70 breaths/min, hepatomegaly, acute moderate and severe malnutrition, and the presence of moderate or severe pallor have been shown to be independently associated with pneumonia-related mortality among children and may be candidate predictors in future risk assessment tools [36,37]. Both the RISC and RISC-Malawi scores demonstrated that the presence of wheezing was associated with lower mortality rates among children, likely suggestive of the presence of a viral etiology, such as bronchiolitis [38]. Moreover, serum lactate and biomarkers such as C-reactive protein and procalcitonin may be associated with severe outcomes in childhood pneumonia [39-42] and may be considered for implementation in future mortality risk assessment tools in settings where these tests are available. However, such biomarkers may not be widely available, could prove cost prohibitive, and involve mildly invasive blood draws.

### Limitations

Though this is the first broad external validation outside of sub-Saharan Africa of the RISC, RISC-Malawi, and PERCH scores, our analysis is subject to limitations. First, the PREPARE data set includes data from studies around the world that were conducted for varying reasons and not necessarily for the external application of these hospitalized pneumonia-related mortality clinical prediction rules. This led to missing variables for many children which precluded our ability to include children with missing data in our external application of these risk scores. As a result, the ROC curves for each score were applied to different patient populations with varying sample sizes. We were not able to apply the RISC score for HIV-positive children to this diverse data set due to lack of reporting of HIV status. This may be an artifact of the enrollment protocols of the studies included in our data set. A large proportion of patients included in our external application of these scores were from Malawi and South Africa, which may have over- or under-estimated the performance of these scores in settings outside of sub-Saharan Africa. Furthermore, we did not control for patient skin color or altitude, which may affect the accuracy of pulse oximetry [43-45].

In terms of the PERCH score, we were unable to externally validate this tool as its developers intended because the WHO PREPARE data set did not include a variable for deep breathing, which may have led to some mis-calibration of our external application of the PERCH risk score. Furthermore, we were only able to apply the PERCH score to 732 children who had complete data for the variables included. Additionally, we only analyzed hospitalized pneumonia-related mortality as post-discharge mortality was not available in the PREPARE data set and the PERCH score predicts hospital and up to 7-day post-discharge mortality. However, post-discharge mortality accounted for only 7/120 (5.8%) of the deaths in the PERCH study. Lastly, we were unable to assess for collinearity of other variables that may have contributed to hospitalized pneumonia-related mortality, as children admitted to hospitals in resource-limited settings often carry more than one diagnosis concomitantly, which could have driven mortality.

## CONCLUSIONS

In a large external application of the RISC, RISC-Malawi, and PERCH scores, a substantial number of children were misclassified for their risk of hospitalized pneumonia-related mortality. The RISC score for HIV-negative children and the PERCH score had poor discriminatory value. The RISC-Malawi score had fair discriminatory value in identifying children at risk of hospitalized pneumonia-related mortality. Further development of a risk assessment tool with greater sensitivity and specificity to identify children at risk of hospitalized pneumonia-related mortality may be warranted. However, careful consideration is needed on whether clinical prediction rules for hospitalized pneumonia-related mortality should aim to be region-specific, considering local epidemiology and resources, or universal. Finally, risk assessment tools must be reassessed over time particularly with the advent of new interventions such as vaccines and medications, and as health systems strengthen and are more able to provide pulse oximetry measurement, supplemental oxygen, and antibiotics.

## Additional material


Online Supplementary Document
