# Supplementary material for: External validation of the RISC, RISC-Malawi, and PERCH clinical prediction rules to identify risk of death in children hospitalized with pneumonia
Source: J Glob Health. 2021 Oct 9;11:04062. doi: 10.7189/jogh.11.04062 (PMC8542381; doi:10.7189/jogh.11.04062)
Supplement: Online Supplementary Document [file jogh-11-04062-s001.pdf]

Online Supplementary Document

Table S1. Number of patients, deaths, case fatality ratio, sensitivity, specificity, correctly classified, positive likelihood ratio (LR+) and negative likelihood ratio (LR-) for Respiratory Index of Severity in Children (RISC) score for HIV-negative children **0-24 months old** in the Pneumonia REsearch Partnerships to Assess WHO REcommendations (PREPARE) dataset (n=3,574)

Score	Patients, n	Deaths, n	Case fatality ratio %[a]	Correctly classified %[b]	Sensitivity[b]	Specificity[b]	LR+ (95% Confidence Interval)[b]	LR- (95% Confidence Interval)[b]
-2	73	2	2.7	1.7	100.0%	0.0%	1.00 (--)	
-1	15	1	6.7	3.6	96.7%	2.0%	0.99 (0.94-1.03)	1.32 (0.41-6.46)
0	862	7	0.8	4.0	94.1%	2.4%	0.98 (0.92-1.03)	1.65 (0.66-6.25)
1	521	7	1.3	27.7	81.6%	26.8%	1.16 (1.02-1.28)	0.59 (0.35-1.08)
2	1,231	10	0.8	41.9	74.1%	41.4%	1.26 (1.05-1.44)	0.64 (0.45-1.01)
3	551	16	2.9	75.8	55.7%	76.1%	2.36 (1.85-2.94)	0.57 (0.44-0.77)
4	241	13	5.4	90.3	29.5%	91.4%	3.64 (2.29-5.12)	0.77 (0.66-0.91)
5	70	4	5.7	96.3	8.2%	97.9%	4.16 (1.61-9.16)	0.93 (0.87-1.01)
6	10	1	10.0	98.1	1.6%	99.7%	5.96 (0.82-49.7)	0.99 (0.95-1.02)

Area under receiver operating curve: 0.66 (95% Confidence Interval 0.58-0.73)

[a] Calculated at each respective score.

[b] Calculated at $\geq$ each respective score.

Table S2. Number of patients, deaths, and case fatality ratio of parameters used in the Respiratory Index of Severity in Children (RISC) score for HIV-negative children expanded to children **0-59 months old** in the Pneumonia REsearch Partnerships to Assess WHO REcommendations (PREPARE) dataset (n=4,061)

**Parameter**	**Sign or symptom present, n (%)**	**Deaths, n**	**Case fatality ratio % (95% CI)**
Oxygen saturation (SpO_2) $\leq$ 90%	988 (24.3)	27	2.7 (1.8-3.9)
Chest indrawing (with SpO_2 >90%)	2,698 (66.4)	31	1.1 (0.8-1.6)
Wheezing	1,677 (41.3)	21	1.2 (0.8-1.9)
Refusal to feed	393 (9.7)	20	5.1 (3.1-7.7)
World Health Organization (WHO) weight for age z-score $\leq$ -3	328 (8.1)	16	4.9 (2.8-7.8)
WHO weight for age z-score -2 $\leq$ z < -3	522 (12.8)	13	2.5 (1.3-4.2)

Table S3. Number of patients, deaths, case fatality ratio, sensitivity, specificity, correctly classified, positive likelihood ratio (LR+) and negative likelihood ratio (LR-) for Respiratory Index of Severity in Children (RISC) score for HIV-negative children expanded to children **0-59 months old** in the Pneumonia REsearch Partnerships to Assess WHO REcommendations (PREPARE) dataset (n=4,061)

Score	Patients, n	Deaths, n	Case fatality ratio %[a]	Correctly classified %[b]	Sensitivity[b]	Specificity[b]	LR+ (95% Confidence Interval)[b]	LR- (95% Confidence Interval)[b]
-2	92	2	2.2	1.6	100.0%	0.0%	1.00	
-1	19	1	5.3	3.8	97.0%	2.2%	0.99 (0.95-1.04)	1.32 (0.33-5.27)
0	1,008	8	0.8	4.2	95.5%	2.7%	0.98 (0.93-1.03)	1.66 (0.54-5.08)
1	570	7	1.2	28.7	83.6%	27.7%	1.16 (1.04-1.29)	0.59 (0.34-1.02)
2	1,374	11	0.8	42.3	73.1%	41.8%	1.26 (1.08-1.46)	0.64 (0.43-0.95)
3	633	17	2.7	75.6	56.7%	76.0%	2.36 (1.90-2.93)	0.57 (0.43-0.75)
4	273	15	5.5	90.4	31.3%	91.4%	3.64 (2.52-5.26)	0.75 (0.64-0.88)
5	81	5	6.2	96.4	9.0%	97.8%	4.16 (1.88-9.18)	0.93 (0.86-1.00)
6	11	1	9.1	98.1	1.5%	99.7%	5.96 (0.77-45.9)	0.99 (0.96-1.02)

Area under receiver operating curve: 0.66 (95% Confidence Interval: 0.59-0.74)

[a] Calculated at each respective score.

[b] Calculated at $\geq$ each respective score.

Table S4. Number of patients, deaths, case fatality ratio, sensitivity, specificity, correctly classified, positive likelihood ratio (LR+), and negative likelihood ratio (LR-) for Respiratory Index of Severity in Children (RISC)-Malawi score with weight-for-age (WAZ) for children 2-59 months old in the PREPARE dataset (n=17,864)

Score	Patients, n	Deaths, n	Case fatality ratio %[a]	Correctly classified %[b]	Sensitivity[b]	Specificity[b]	LR+ (95% Confidence Interval)[b]	LR- (95% Confidence Interval)[b]
-1	1,102	12	1.1	4.9	100.0%	0.0%	1.00	--
0	3,664	45	1.2	10.9	98.6%	6.4%	1.05 (1.04-1.06)	0.21 (0.12-0.38)
1	3,563	54	1.5	30.9	93.4%	27.7%	1.29 (1.27-1.32)	0.24 (0.18-0.30)
2	1,107	34	3.1	50.2	87.2%	48.4%	1.69 (1.64-1.74)	0.26 (0.22-0.31)
3	964	27	2.8	56.1	83.3%	54.7%	1.84 (1.78-1.90)	0.30 (0.26-0.35)
4	1,181	44	3.7	61.2	80.2%	60.2%	2.01 (1.94-2.09)	0.33 (0.29-0.38)
5	1,582	118	7.5	67.3	75.1%	67.9%	2.27 (2.17-2.37)	0.37 (0.33-0.42)
6	1,768	158	8.9	74.8	61.6%	75.5%	2.51 (2.37-2.66)	0.51 (0.47-0.55)
7	775	58	7.5	82.9	43.4%	85.0%	2.88 (2.65-3.14)	0.67 (0.63-0.71)
8	632	69	10.9	86.6	36.7%	89.2%	3.39 (3.08-3.74)	0.71 (0.67-0.75)
9	345	50	14.5	89.4	28.8%	92.5%	3.83 (3.41-4.31)	0.77 (0.74-0.80)
10	258	36	14.0	90.8	23.0%	94.2%	3.99 (3.48-4.57)	0.82 (0.79-0.85)
11	527	90	17.1	91.8	18.9%	95.5%	4.23 (3.62-4.93)	0.85 (0.82-0.88)
12	284	43	15.1	93.7	8.5%	98.1%	4.49 (3.52-5.73)	0.93 (0.91-0.95)
13	38	9	23.7	94.9	3.6%	99.5%	7.48 (4.98-11.30)	0.97 (0.96-0.98)
14	14	5	35.7	95.0	2.5%	99.7%	8.27 (5.05-13.60)	0.98 (0.97-0.99)
15	15	3	20.0	95.0	2.0%	99.7%	7.73 (4.43-13.50)	0.98 (0.97-0.99)
16	32	11	34.4	95.0	1.6%	99.8%	8.83 (4.72-16.50)	0.99 (0.98-0.99)
17	13	3	23.1	95.1	0.3%	99.9%	5.87 (1.62-21.30)	1.00 (0.99-1.00)

Area under receiver operating curve: 0.75 (95% Confidence Interval: 0.74-0.77)

[a] Calculated at each respective score.

[b] Calculated at $\geq$ each respective score.

Table S5. Number of patients, deaths, and case fatality ratio of parameters used in the Respiratory Index of Severity in Children (RISC)-Malawi (middle upper arm circumference [MUAC]) score for children 2-59 months old in the two datasets in the Pneumonia REsearch Partnerships to Assess WHO REcommendations (PREPARE) dataset that included all 5 parameters (n=2,251)

**Parameter**	**Weighted score**	**Patients, n**	**Deaths, n**	**Case fatality ratio % (95% Confidence Interval)**
**Oxygen saturation (SpO_2)**				
SpO_2 ≥93%	0	1,440 (64.0)	66	4.6 (3.6-5.8)
SpO_2 90-92%	2	407 (18.1)	26	6.4 (4.2-9.2)
SpO_2 <90%	7	404 (17.9)	59	14.6 (11.3-18.4)
**MUAC**				
MUAC >13.5 cm	0	626 (27.8)	19	3.0 (1.8-4.7)
MUAC 11.5-13.5 cm	3	1,093 (48.5)	67	6.1 (4.8-7.7)
MUAC <11.5 cm	7	532 (23.6)	65	12.2 (9.6-15.3)
**Female**	1	781 (34.7)	52	6.7 (5.0-8.6)
**Wheezing**	-2	1,064 (47.3)	50	4.7 (3.5-6.1)
**Unconscious/decreased consciousness**	8	134 (5.9)	9	6.7 (3.1-12.4)

Table S6. Application of the Respiratory Index of Severity in Children (RISC)-Malawi (MUAC) score to children 2-59 months of age in studies including all 5 parameters in the Pneumonia REsearch Partnerships to Assess WHO REcommendations (PREPARE) dataset (n=2,251)

Score	Patients, n	Deaths, n	Case fatality ratio %[a]	Correctly classified %[b]	Sensitivity [b]	Specificity [b]	LR+ (95% Confidence Interval)[b]	LR- (95% Confidence Interval)[b]
-2	121	1	0.8	6.7	100.0%	0.0%	1.00	
-1	56	1	1.8	12.0	99.3%	5.7%	1.05 (1.04-1.07)	0.12 (0.02-0.82)
0	147	2	1.4	14.4	98.7%	8.3%	1.08 (1.05-1.10)	0.16 (0.04-0.63)
1	389	13	3.3	20.7	97.3%	15.2%	1.15 (1.11-1.19)	0.17 (0.07-0.46)
2	118	2	1.7	36.9	88.7%	33.1%	1.33 (1.24-1.42)	0.34 (0.22-0.53)
3	300	14	4.7	41.9	87.4%	38.7%	1.43 (1.33-1.53)	0.32 (0.21-0.50)
4	148	10	6.8	54.0	78.1%	52.3%	1.64 (1.49-1.80)	0.42 (0.31-0.57)
5	169	12	7.1	59.7	71.5%	58.9%	1.74 (1.55-1.95)	0.48 (0.37-0.62)
6	69	8	11.6	66.1	63.6%	66.3%	1.89 (1.65-2.16)	0.55 (0.44-0.68)
7	148	14	9.5	68.5	58.3%	69.2%	1.89 (1.63-2.20)	0.60 (0.50-0.73)
8	179	16	8.9	73.8	49.0%	75.6%	2.01 (1.68-2.40)	0.67 (0.58-0.79)
9	90	8	8.9	80.4	38.4%	83.4%	2.31 (1.85-2.89)	0.74 (0.65-0.84)
10	77	14	18.2	83.6	33.1%	87.3%	2.60 (2.02-3.35)	0.77 (0.68-0.86)
11	48	6	12.5	85.8	23.8%	90.3%	2.45 (1.79-3.36)	0.84 (0.77-0.92)
12	58	4	6.9	87.4	19.9%	92.3%	2.58 (1.81-3.67)	0.87 (0.80-0.94)
13	15	1	6.7	89.6	17.2%	94.9%	3.35 (2.26-4.97)	0.87 (0.81-0.94)
14	57	14	24.6	90.2	16.6%	95.5%	3.70 (2.46-5.57)	0.87 (0.81-0.94)
15	30	8	26.7	91.5	7.3%	97.6%	3.00 (1.60-5.63)	0.95 (0.91-0.99)
16	14	1	7.1	92.1	2.0%	98.6%	1.44 (0.44-4.67)	0.99 (0.97-1.02)
18	6	0	0	92.7	1.3%	99.2%	1.74 (0.40-7.49)	0.99 (0.98-1.01)
19	7	0	0	92.9	1.3%	99.5%	2.78 (0.61-12.6)	0.99 (0.97-1.01)
22	3	2	66.7	93.2	1.3%	99.9%	9.27 (1.56-55.1)	0.99 (0.97-1.01)
23	2	0	0	93.2	0.0%	99.9%	0.00 (--)	1.00 (1.00-1.00)

Area under receiver operating curve: 0.70 (95% Confidence Interval, 0.66-0.74)

[a] Calculated at each respective score.

[b] Calculated at $\geq$ each respective score.

Table S7. Number of patients, deaths, case fatality ratio, correctly classified, sensitivity, specificity, positive likelihood ratio (LR+) and negative likelihood ratio (LR-) for Pneumonia Etiology Research for Child Health (PERCH) Score for HIV-negative children **1-59 months old** in the Pneumonia REsearch Partnerships to Assess WHO REcommendations (PREPARE) dataset (n=732)

Score	Patients, n	Deaths, n	Case fatality ratio %[a]	Correctly classified %[b]	Sensitivity[b]	Specificity[b]	LR+ (95% Confidence Interval)[b]	LR- (95% Confidence Interval)[b]
-1	5	0	0.0	2.2	100.0%	0.0%	--	--
0	4	0	0.0	2.9	100.0%	0.7%	1.01 (1.00-1.01)	--
1	27	1	3.7	3.4	100.0%	1.3%	1.01 (1.00-1.02)	--
2	18	1	5.6	6.8	93.7%	4.9%	0.99 (0.87-1.12)	1.28 (0.19-8.77)
3	94	2	2.1	9.0	87.5%	7.3%	0.94 (0.78-1.14)	1.72 (0.46-6.46)
4	70	2	2.9	21.3	75.0%	20.1%	0.94 (0.71-1.25)	1.24 (0.52-2.94)
5	172	1	0.6	30.3	62.5%	29.6%	0.89 (0.61-1.30)	1.27 (0.67-2.41)
6	123	2	1.6	53.5	56.2%	53.5%	1.21 (1.78-1.88)	0.82 (0.47-1.43)
7	93	1	1.1	69.8	43.7%	70.4%	1.48 (0.84-2.60)	0.80 (0.52-1.23)
8	73	3	4.1	82.2	37.5%	83.2%	2.24 (1.16-4.30)	0.75 (0.51-1.10)
9	26	1	3.9	91.4	18.7%	93.0%	2.69 (0.93-7.71)	0.87 (0.69-1.11)
10	18	1	5.6	94.7	12.5%	96.5%	3.58 (0.93-13.80)	0.91 (0.75-1.09)
11	6	1	16.7	96.9	6.2%	98.9%	5.59 (0.74-42.1)	0.95 (0.84-1.08)
12	3	0	0.0	97.4	0.0%	99.6%	--	1.00 (1.00-1.01)

Area under receiver operating curve: 0.55 (95% Confidence Interval, 0.37-0.73)

[a] Calculated at each respective score.

[b] Calculated at $\geq$ each respective score.
